# Seven-Year Single-Center Experience of the Efficacy and Safety of Ferric Carboxymaltose in Cancer Patients with Iron-Deficiency Anemia

**DOI:** 10.3390/curroncol30110703

**Published:** 2023-11-02

**Authors:** Burak Yasin Aktaş, Emine Büşra Ata, Engin Çeşmeci, İbrahim Yahya Çakır, Muharrem Coşkunpınar, Yağmur Tahillioğlu, Gürkan Güner, Deniz Can Güven, Zafer Arık, Neyran Kertmen, Ömer Dizdar, Şuayib Yalçın, Sercan Aksoy

**Affiliations:** 1 Department of Medical Oncology, Hacettepe Cancer Institute, Ankara 06230, Turkey; guner.gurkan@hacettepe.edu.tr (G.G.); deniz.can.guven@hacettepe.edu.tr (D.C.G.); zafer.arik@hacettepe.edu.tr (Z.A.); neyran.kertmen@hacettepe.edu.tr (N.K.); omer.dizdar@hacettepe.edu.tr (Ö.D.); syalcin@hacettepe.edu.tr (Ş.Y.); 2 Department of Medical Oncology, Guy’s and Saint Thomas’ NHS Trust, London SE1 9RT, UK; 3 Department of Internal Medicine, Faculty of Medicine, Hacettepe University, Ankara 06230, Turkey; busraata@hacettepe.edu.tr (E.B.A.); engincesmeci@hacettepe.edu.tr (E.Ç.); ibrahimyahyacakir@hacettepe.edu.tr (İ.Y.Ç.); muharremcoskunpinar@hacettepe.edu.tr (M.C.); yagmur.tahillioglu@hacettepe.edu.tr (Y.T.)

**Keywords:** iron deficiency, ferric carboxymaltose, cancer-related anemia

## Abstract

Anemia remains an essential concern affecting the quality of life and the survival of cancer patients. Although there are different approaches to treating anemia in cancer patients, the number of studies reporting the efficacy of iron replacement in cancer patients is limited. In this study, the efficacy and safety of iron carboxymaltose, a parenteral iron treatment option, in the treatment of anemia, were examined retrospectively. A total of 1102 adult patients who received IV ferric carboxymaltose treatment at Hacettepe Oncology Hospital between 2014 and 2020 were included. The mean hemoglobin change observed at the end of the 12th week was 1.8 g/dL, and the rate of patients with an increase in hemoglobin of 1 g/dL or more was 72.1%. It was observed that the treatment demonstrated effectiveness in patients receiving active cancer treatment in all tumor types. The treatment was generally safe, and no grade 3–5 side effects were observed in the patients included in the study. According to one of the most extensive series published in the literature, iron carboxymaltose is an efficient and safe alternative for cancer patients with iron-deficiency anemia.

## 1. Introduction

Anemia is the most common hematological disorder in cancer patients. The prevalence of anemia among cancer patients exhibits variability across various studies. In a prospective European epidemiological survey including 15,000 cancer patients, anemia was present in approximately 40% of patients at baseline, and the overall prevalence increased to 67% during a six-month follow-up [1]. Anemia is a prevalent contributor to the fatigue experienced by individuals with cancer, and it can also have negative effects on treatment compliance, resulting in diminished therapeutic outcomes, prolonged hospitalization, and potentially reduced survival rates [2]. One of the formidable issues encountered in routine oncology practice is the occurrence of anemia, which leads to the necessity of reducing or postponing intended chemotherapy dosages for the treatment of both early and advanced-stage malignancies [3]. Moreover, a systemic meta-analysis reported a 65% increased mortality risk in cancer patients with anemia [4]. On the other hand, treating anemia has been shown to improve fatigue, quality of life, and performance scores [5].

The development of anemia in cancer patients is a complex process and is generally multifactorial. The leading etiologies of anemia in cancer patients encompass various factors, notably the insufficiency of micronutrients including iron and vitamin B12, the presence of anemia of chronic disease associated with elevated pro-inflammatory cytokines, impairments in erythropoiesis due to cytotoxic agents, tumor infiltration of the bone marrow, ongoing occult or overt hemorrhages, and intravascular hemolysis [6].

Iron deficiency is one of the most prevalent causes of anemia in cancer patients, with reported rates ranging from 30% to 40% across different studies [7]. It has also been shown that anemia is related to the disease stage and the patient’s performance status [8,9].

There are many factors that contribute to the emergence of iron deficiency during the malignant process. One major reason of iron deficiency is decreased iron intake, which is common in cancer patients. Several factors contribute to this phenomenon, including the development of anorexia as a result of elevated levels of pro-inflammatory cytokines like TNF alpha, or in more severe cases, cachexia associated with cancer. Additionally, the presence of a combination of drugs can lead to compromised intestinal mucosa and impaired nutrient absorption. Furthermore, gastrointestinal tract obstructions may also be part of the picture [10,11]. Increased iron loss due to bleeding, which constitutes an important trigger in the occurrence of iron insufficiency, is commonly reported across several types of malignancies, particularly those affecting the gastrointestinal system.

Disparities exist in the characterization of iron-deficiency anemia between individuals with cancer and the general population. There are multiple possible explanations for this occurrence. Anemia in cancer patients can be influenced by multiple underlying factors [10]. Additionally, the serum iron parameters commonly employed to diagnose iron deficiency are acute phase reactants, suggesting that they can be significantly impacted by the presence of cancer itself. While the hemoglobin cutoff values established by the World Health Organization (WHO) are considered genuine, numerous studies have defined the threshold for anemia in cancer patients as 12 g/dL. Iron deficiency can manifest in cancer patients as either absolute iron deficiency, characterized by low levels of ferritin in conjunction with reduced transferrin saturation, or more commonly, as functional iron deficiency with normal or high levels of ferritin. The assessment of soluble transferrin receptor and hepcidin has emerged as a promising diagnostic approach for detecting and distinguishing iron deficiency, with the potential for incorporation into standard clinical protocols in the foreseeable future [10,12,13].

The management of iron-deficiency anemia in cancer patients presents many obstacles. While handicaps such as obstructions affecting absorption, concomitant drug use, or GI intolerance limit the use of oral preparations, infusion reactions, recurrent hospital visits, and an increased frequency of infections complicate the use of intravenous agents. Oral iron preparations remain controversial in terms of treatment compliance and efficacy, and their use is not recommended except for a minor group of patients with absolute iron-deficiency anemia who can tolerate treatment [14]. Ferric carboxymaltose has been identified as a promising therapeutic agent for addressing the challenges associated with treating iron deficiency in cancer patients. It is a dextran-free iron preparation that has shown efficacy and safety in phase 3 studies in numerous diseases, such as inflammatory bowel disease, chronic renal failure, and heart failure [15,16,17]. In oncology practice, a limited number of previous studies have shown the effectiveness of this treatment, especially among perioperative patient groups [18].

However, there is a scarcity of both prospective and retrospective research assessing the efficacy of iron carboxymaltose treatment in cancer patients with iron-deficiency anemia. The number of patients in the studies reported so far is limited, and more data are needed to address this prevalent clinical problem. Additionally, there has yet to be previous data reported from Turkey. In order to point out this gap in the literature, we aimed to retrospectively examine cancer patients who received iron carboxymaltose treatment for iron-deficiency anemia over seven years at Hacettepe Oncology Hospital, one of the largest comprehensive cancer centers in Turkey. The high patient volume at this hospital and the routine use of iron carboxymaltose therapy in patients with iron-deficiency anemia allowed for the analysis of treatment efficacy in subgroups, including different tumor types and active anti-cancer treatment status.

## 2. Materials and Methods

In this single-center retrospective study, we investigated the efficacy and safety of ferric carboxymaltose treatment among patients with cancer. The study included cancer patients over 18 years old who received at least one dose of ferric carboxymaltose for iron-deficiency anemia treatment between 1 January 2014 and 31 December 2020 at Hacettepe Oncology Hospital.

This study was approved by the non-interventional institutional review board of Hacettepe University (GO22/135, 10 January 2023).

Patient records and relevant information were obtained from the electronic hospital database. Patient characteristics, including age, gender, tumor type, treatment status, and baseline hematological parameters, including complete blood count, ferritin level, transferrin saturation, and the 12th-week hemoglobin value, were recorded.

Iron-deficiency anemia is defined as a hemoglobin value below 12 g/dL, along with a low ferritin level (<30 ng/mL) or transferrin saturation (20%). Patients who received RBC transfusions within 12 weeks after ferric carboxymaltose treatment with a baseline hemoglobin level of 12 g/dL or above or who did not have a 12th-week hemoglobin level, were excluded.

Efficacy was assessed by the delta (Δ) hemoglobin value and the response rate to IV iron treatment. Delta hemoglobin was the difference between the hemoglobin values at week 12 and baseline. Treatment response was defined as an increase in hemoglobin of 1 g/dL or more after treatment. 

Efficacy was examined in the overall group and subgroups. Subgroups were determined by gender, age category (geriatric vs. non-geriatric), tumor type, anemia grade, and active anti-cancer treatment status. Due to the high number of patients, gastrointestinal tumors were examined separately as a cohort. Descriptive and inferential statistical evaluation was also performed on subgroups of this cohort.

Safety was assessed by analyzing the incidence and severity of infusion reactions and post-treatment adverse events (AEs) as recorded by the treating physician.

All statistical analyses were performed using SPSS software version 22.0. We used visual methods, including histograms and probability plots, and analytic methods, such as the Kolmogorov–Smirnov or Shapiro–Wilk’s test, to determine if the variables were normally distributed. Categorical data are summarized as numbers and percentages. Continuous variables are presented as mean ± standard deviation unless otherwise stated. We examined the relationship between different clinical variables and treatment response parameters (Δ hemoglobin and response rate). We used the Chi-square or Fisher exact tests for categorical data, and independent-sample *t*-tests or ANOVA for continuous data. A two-sided *p*-value < 0.05 was considered statistically significant.

## 3. Results

Between January 2014 and December 2020, 1328 cancer patients received iron carboxymaltose for the treatment of iron-deficiency anemia at Hacettepe Oncology Hospital adult oncology clinics. A total of 122 patients were excluded from the study due to exceeding the pre-defined baseline hemoglobin level (12 g/dL), 90 patients were excluded due to missing data, including baseline hematological parameters and/or 12th-week complete blood count, and 14 were excluded due to receiving red blood cell transfusions within the 12-week period. A total of 1102 patients were included for further analysis.

The median age of the group was 60 (48–68), and 696 of patients (63.2%) were female. Of the patients, 46.3% (N = 510) had gastrointestinal system cancer, and 28.9% had breast cancer (N = 319). Most patients (78.9%) received systemic anti-cancer therapy within 12 weeks after iron therapy. The median dose of iron carboxymaltose administered was 1000 mg (500–2000 mg). The proportion of patients with grade 1, 2, and 3 anemia was 69.3%, 27.8% and 2.9%, respectively. The baseline characteristics of the patients are shown in Table 1.

The mean baseline hemoglobin level of the group was 10.4 g/dL (6.6–11.9 g/dL). The baseline median ferritin was 23.7 ng/mL, and the median transferrin saturation was 8%. The mean hemoglobin level at the 12th week after treatment was 12.2 g/dL (7.8–16.0 g/dL), while the average hemoglobin change (Δ hemoglobin) was 1.8 (±0.14) g/dL. Baseline and follow-up hematological parameters are presented in Table 2.

**Figure 1:**
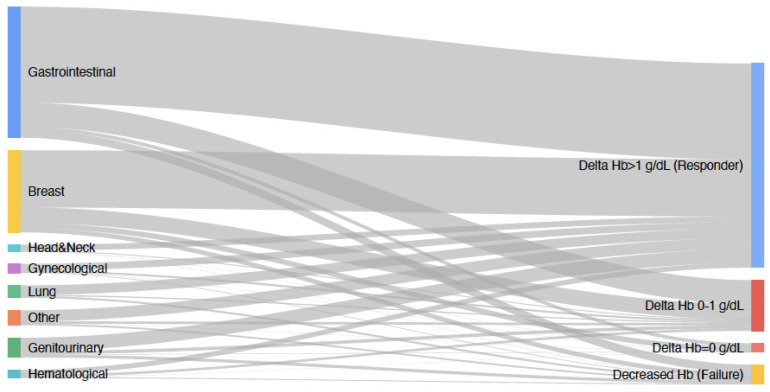
Post-treatment hemoglobin changes according to tumor types.

A 1.0 g/dL or greater increase in hemoglobin value (treatment response) was observed among 794 patients (72.1%). Of the group, 17.9% showed an increase in hemoglobin below 1 g/dL. At the 12th-week follow-up, no hemoglobin changes were observed in 3.1% of the patients, while a hemoglobin decrease (treatment failure) was noted in 7.0%. The post-treatment change in hemoglobin according to tumor types is shown in Figure 1.

Regarding sub-group analysis, no difference was observed in Δ hemoglobin between male and female patients (*p* = 0.87) or between geriatric and non-geriatric patient groups (*p* = 0.35). There was no significant difference in Δ hemoglobin between tumor types (*p* = 0.67). Among patients with grade 1, 2, and 3 anemia, the hemoglobin increase was detected as 1.5 g/dL, 2.4 g/dL, and 3.0 g/dL, respectively (*p* < 0.005). The mean Δ hemoglobin in patients not receiving active systemic cancer treatment was 2.0 g/dL, while it was 1.7 g/dL in those receiving active treatment (*p* = 0.005). Although this difference appears to be statistically significant, clinically, it is not.

A 1 g/dL or more increase in hemoglobin was considered a treatment response, attributing the expected contribution of a 1-unit RBC transfusion. A total of 794 patients (72.1%) were responsive to treatment. A significantly higher response rate was observed among female patients compared to male patients (74.5% vs. 67.7%, *p* = 0.018). No significant difference was found in age groups, type of underlying malignancy, or anti-cancer treatment status. A comparison of treatment efficacy parameters in subgroups is shown in Table 3.

A total of 510 patients with gastrointestinal system cancer were included in the study. The median age of the group was 57 (45–68), and 252 (49.4%) of the patients were female. The number of patients with upper GI (gastroesophageal), HPB (hepato-pancreato-biliary), and lower GI (colorectal) cancers was 147, 80, and 282, respectively. The majority of patients were receiving active anti-cancer treatment (N = 423, 82.9%). The mean baseline hemoglobin of the patients was 10.4 (±1.7) g/dL, and the mean Δ hemoglobin was 1.76 (±0.34) g/dL. The proportion of patients with a treatment response was 72.9%. Baseline characteristics of GI cancer patients are presented in Table 4.

In the GI tumor cohort, ferric carboxymaltose treatment response was independent of age (*p* = 0.54) and gender (*p* = 0.21). Additionally, no significant difference was shown in the mean Δ hemoglobin value between male and female patients (*p* = 0.81) or between geriatric and non-geriatric groups (*p* = 0.87). The mean delta hemoglobin value was 1.80, 1.78, and 1.74 in the upper GI, HPB, and lower GI groups, respectively. There was no significant difference between GI tumor locations in terms of response rates (*p* = 0.4). Both the mean Δ hemoglobin (1.71 vs. 2.04, *p* = 0.03) and the pre-defined treatment response rate (72.9% vs. 84.0%, *p* = 0.04) were worse in patients receiving active treatment compared to those not on treatment. Comparison of treatment efficacy parameters among GI cancer subgroups is shown in Table 5.

In the safety assessment of all groups, a total of 4 patients had infusion reactions. Among these four ferric carboxymaltose-related infusion reactions, three were Grade 1, and one was Grade 2. Three patients reported G1 pruritus after treatment. No G3-5 adverse events were reported.

## 4. Discussion

This study retrospectively examined the efficacy and safety of ferric carboxymaltose among cancer patients with iron-deficiency anemia treated at a tertiary reference cancer center. To our knowledge, our study is the most extensive retrospective series reported to date.

The mean hemoglobin change (Δ hemoglobin) at the 12th week after the first dose of ferric carboxymaltose treatment was 1.8 g/dL. There are different series in which hemoglobin changes were monitored after both oral and intravenous iron replacement in oncology patients. In a prospective, non-invasive multicenter pilot study, the mean Δ hemoglobin was 1.3 g/dL in 277 cancer patients treated with ferric carboxymaltose [19]. In a single-center phase 2 study including 92 patients receiving active anti-cancer treatment, the mean Δ hemoglobin value at the 8th week was reported as 1.77 g/dL, and the responder patient rate was 66.5% [20]. In a prospective observational study reported from France, the mean hemoglobin change in 236 cancer patients in the third month after ferric carboxymaltose treatment was 1.5 g/dL [21]. All three of these studies showed very similar efficacy of ferric carboxymaltose compared to our study. In our analysis, ferric carboxymaltose treatment shows efficacy at the 12th week in a large retrospective series of 1102 patients.

On the other hand, Verhaeghe et al. reported a retrospective analysis of 303 gastrointestinal cancer patients who had ferric carboxymaltose treatment. In this study, Δ hemoglobin at week 4 was 0.5 g/dL [22]. It can be argued that the lower Δ hemoglobin reported in this trial is related to the short time interval between baseline and follow-up measurements. Makharadze et al. recently reported, in an IRON-CLAD phase 3 randomized placebo-controlled ferric carboxymaltose trial that included 244 actively treated cancer patients, that the rate of patients who had an increased hemoglobin (Hb) level of ≥0.5 g/dL between the 3rd and 18th week (primary endpoint) was significantly higher in the intervention arm (50.8% vs. 35.3%; *p* = 0.01). Even though the primary endpoint was chosen to be ≥0.5 g/dL in this study, the response rate was lower than our results. However, in a secondary study analysis, the rate of patients with an increase of 1 g/dl or more at week 18 was reported to be 70.6% [23].

A 1 g/dL or more increase in hemoglobin was found in 72.1% of the 1102 patients after iron carboxymaltose treatment. An increase of 1 g/dL in hemoglobin level has also been defined as a treatment response in numerous other series. It should also be noted that an increase in hemoglobin, even below 1 g/dL, was observed in 17.9% of the patients, and only 7% had anemia that deepened after treatment. Primary unresponsiveness to treatment may be due to multifactorial anemia etiology or ongoing iron loss in patients.

In a recently published prospective study, the effectiveness of ferric carboxymaltose treatment was examined in 84 anemia patients. In this study, patients were divided into “absolute iron deficiency”, “functional iron deficiency” and “other” (TSAT > 20%) groups according to ferritin and transferrin saturation. The overall treatment response rate was 59.5%, while a difference was noted between the absolute and functional iron deficiency groups (80.8% vs. 70.8%). Although the results of the study indicate that the effectiveness may be lower in patients with functional iron-deficiency anemia, treatment success is still high in both groups and consistent with our findings [24].

In a randomized controlled trial conducted in a perioperative colon cancer cohort, patients were assigned to ferric carboxymaltose and oral iron treatment arms two weeks before surgery. The primary endpoint of the study was determined as the rate of patients whose hemoglobin level was normalized. While no difference was observed between the groups on the second week, the response rate was reported to be 60% in the intravenous arm and 21% in the oral treatment arm on day 30 [25]. The underlying reason for the worse response rate in this study compared to our analysis may be that the patients underwent colon surgery during the study period. However, intravenous treatment appears to be more effective than oral treatment. There are also studies examining other intravenous preparations. In one of the early studies evaluating iron sucrose treatment, 25 cancer patients were treated, and the rate of patients with hemoglobin elevation of 1 g/dL and above was reported as 42.1% [26]. A recent single-center, randomized, controlled trial showed similar delta hemoglobin results among post-operative colon cancer patients who received ferric carboxymaltose or iron sucrose (2.5 g/dL vs. 2.4 gr/dL, *p* = 0.52). Iron sucrose treatment was also associated with higher infection rates (37.2% vs. 9.8%) [27]. Besides the response rate, the disadvantages of iron sucrose therapy include that it requires a 12-week treatment schedule and has a worse safety profile.

In the present investigation, it has been observed that the effectiveness of ferric carboxymaltose therapy does not seem to be influenced by the age of the individuals. There was no difference between the geriatric and non-geriatric groups in terms of both Δ hemoglobin and response rate. In a retrospective study investigating the efficacy of treatment in the hospitalized geriatric population over the age of 75, where oncology patients are underrepresented, the response rate was determined by similar criteria, and the response was observed in 18 of 38 patients (47.7%) [28]. The reason for reporting lower response rates in this study may be attributed to the inclusion of fewer patients, being in an older age group, or the fact that the patients were inpatients, a more vulnerable group. In other studies of iron therapy in oncology patients, no analysis of treatment response by age group has been reported. We think that this finding obtained in our study is remarkable.

Our study was conducted at a comprehensive oncology center, and patients with various tumor types were included. However, it should be underlined that a significant portion of the patients were gastrointestinal cancer patients. This may be due to the high prevalence of GI cancers, the high rate of iron deficiency among GI cancer patients, or the tendency to prefer parenteral iron due to reasons including obstruction and ongoing blood loss in GI cancers. Nevertheless, our study included patients who were underrepresented in previous iron-deficiency anemia treatment studies as well. It has been observed that treatment effectiveness occurs in all tumor types. So far, different studies conducted in different tumor groups have shown that treatment effectiveness is independent of histology. This study is consistent with previous studies and also informative about unreported tumor types [19,21].

As the grade of anemia increased, Δ hemoglobin values and response rates increased. This finding is compatible with clinical practice because the Ganzoni formula is used to determine the iron dose to be administered in our hospital [29]. As anemia worsens, a better hemoglobin increase is obtained depending on the increase in the iron dose given. The transfusion rate is beyond the scope of this study; however, the average hemoglobin increase of 3.06 g/dL and the 93.7% response rate seen in 32 patients with grade 3 anemia, who are candidates for transfusion, indirectly indicate that iron carboxymaltose treatment is also effective in profound anemias requiring transfusion. A multi-center observational study has shown that ferric carboxymaltose treatment significantly decreased transfusion rates among patients with colon cancer (9.9 vs. 38.7%; *p* < 0.001) [30]. Another prospective study conducted in Portugal between 2015 and 2016 showed a 26% decrease in the transfusion rate compared to the historical control group [31].

The response rate was 72.9% among patients on active anti-cancer treatment. These findings indicate that iron carboxymaltose is a vital option for preventing iron-deficiency anemia-related anti-tumoral treatment delays and dose reductions. The mean Δ hemoglobin in patients under active anti-cancer treatment was 1.74 g/dL, while it was 2.01 g/dL in those not receiving treatment. Although this difference seems to be statistically significant, clinically, it is not. The fact that this value reached statistical significance is related to the large number of patients included in the study.

Due to the large number of GI tumor patients included in this study, additional analyses were performed in this patient group. The mean Δ hemoglobin in the GI tumor group was 1.76 (±0.34) g/dL and the response rate was 72.9%. The occurrence of recurrent bleeding and difficulties with oral intake are frequently observed in the therapeutic management of gastrointestinal malignancies. As a result, there have been studies conducted on the use of ferric carboxymaltose in this particular patient population, as documented in the existing literature. In a randomized study conducted on pre-operative colorectal cancer patients, the efficacy of oral iron supplementation was compared to IV ferric carboxymaltose. The results indicated that the median Δ hemoglobin in the intravenous (IV) iron group was 1.5 gr/dL, with a corresponding response rate of 90%. The elevated response rate documented in this trial could potentially be attributed to the inclusion of exclusively early stage patients or the absence of exclusion criteria for individuals receiving blood transfusions [18]. In contrast to the overall group, the cohort of GI patients who had active anti-cancer treatment exhibited inferior treatment responses compared to those who did not. Similarly, the mean Δ hemoglobin was 0.5 gr/dL in a retrospective study on ferric carboxymaltose in 303 gastrointestinal cancer patients, 90% of whom received active anticancer therapy [22]. The observed variations may be attributed to variances in the stages of patients or ongoing iron loss due to hemorrhage. Further investigation through prospective research is necessary to explore this topic.

Although the therapy response rate of ferric carboxymaltose is considerable, it has been shown that the intended improvement in hemoglobin levels is not observed in approximately 27% of patients in our study. Additionally, 10.1% of patients experienced either stable or worsening anemia. Given the retrospective nature of this study, it does not encompass additional evaluations of individuals with refractory anemia. Iron loss due to ongoing active or occult bleeding may contribute to persistent anemia among this patient group. The presence of persistent and active inflammation, particularly in individuals at an advanced stage of the disease, may play a crucial role in the development of resistance to intravenous iron replacement. The elevation of hepcidin levels resulting from inflammation has a role in the development of anemia in individuals with cancer. This is attributed to its dual effect of hindering the absorption of iron from the gut and inducing functional iron deficiency through the accumulation of iron in the liver and macrophages [32]. The assessment of anemia of inflammation accompanied by iron deficiency anemia is increasingly incorporating the measurement of serum hepcidin and reticulocyte hemoglobin levels as relevant indicators [33,34]. It has been reported that low hepcidin levels improve iron carboxymaltose treatment success in patients with chronic renal failure [35]. The precise demonstration of this link in cancer patients who received iron carboxymaltose was not achieved [23]. B12 and folate deficiency should also be kept in mind in anemia refractory to iron treatment [36].

Rare grade 1–2 adverse events have been noted with ferric carboxymaltose treatment, and no grade 3–5 side effects were observed in the patients included in this study. These results are consistent with those of previous studies [19,28,37]. The treatment is safe and tolerable. Of note, hypophosphatemia following intravenous ferric carboxymaltose therapy has become an adverse effect that has attracted increasing attention. In a recently published retrospective study, the rate of moderate to severe hypophosphatemia was reported to be 36% in 174 patients receiving ferric carboxymaltose treatment. Unfortunately, since our data series covers many years and early serum biochemistry measurements were not made in many patients, the frequency of hypophosphatemia could not be reported in our study. However, monitoring this potential side effect seems important [38].

Our study has strengths and limitations. Its main strengths are that it reflects the long-term experience of a single center and that it includes the highest number of patients reported so far in the literature. It also represents the real world, including various tumor types and grade 1, 2, and 3 anemic patients. We believe that the results of the study can contribute to the daily practices of other centers that treat iron-deficiency anemia in cancer patients, as it includes real-life data representing the long-term experience of a comprehensive oncology center. Additionally, including individuals with different tumor types, such as head and neck cancer, which has not been reported in previous studies, provides a broader-based evaluation opportunity. The main limitations are the retrospective design of the study and the lack of additional data on long-term follow-up. Additionally, most patients were GI and breast cancer patients, which may make interpreting statistical results difficult. However, this situation is consistent with real life and previous studies. The tendency for anemia is high in gastrointestinal and breast cancers, and these two tumors constitute the main groups included in many previous ferric carboxymaltose studies. Since the data were collected retrospectively, other parameters, including hepcidin and acute phase reactants, were not requested per protocol. Prospective designs in which pre-planned parameters are evaluated may be advantageous in future studies.

## 5. Conclusions

This real-world study of ferric carboxymaltose treatment in cancer patients with iron-deficiency anemia highlights that ferric carboxymaltose is highly efficient and tolerable. Moreover, efficacy is shown in all subgroups regardless of age, tumor type, or active anti-cancer treatment status. However, further prospective studies that encompass particular underrepresented tumor categories are required. Additionally, further research is warranted to investigate the attributes and treatment strategies for patients who are refractory to iron carboxymaltose. In conclusion, ferric carboxymaltose is an appropriate alternative for cancer patients with iron-deficiency anemia.

## Figures and Tables

**Table 1:** Baseline characteristics.

	N = 1102
Age (years, median (Q1–Q3))	60 (48–68)
Female, *n (%)*	696 (63.2%)
Cancer type, *n (%)*	
Gastrointestinal	510 (46.3%)
Breast	319 (28.9%)
Genitourinary	76 (6.9%)
Lung	48 (4.4%)
Gynecological	35 (3.2%)
Hematological	31 (2.8%)
Head and Neck	25 (2.3%)
Other	58 (5.3)
Active chemotherapy, *n (%)*	870 (78.9%)
Anemia Grade *n (%)*	
1	764 (69.3%)
2	306 (27.8%)
3	32 (2.9%)

**Table 2:** Baseline and follow-up hematological parameters.

	N = 1102
Baseline hemoglobin	
Mean (SD)	10.4 (±1.1) g/dL
Median (Q1–Q3)	10.7 (9.7–11.4)
Leucocyte (Median (Q1–Q3))	6200 (4400–8100)
Thrombocyte (Median (Q1–Q3))	295,000 (220,000–382,000)
Ferritin (Median (Q1–Q3))	23.7 (16.7–37.8)
Transferrin saturation (Median (Q1–Q3))	8 (5–12)
Week-12 hemoglobin	
Mean (SD)	12.2 (±1.4) g/dL
Median (Q1–Q3)	12.3 (11.3–13.3)
Δ hemoglobin	
Mean (SD)	1.81 (±0.14) g/dL
Median (Q1–Q3)	1.60 (0.8–2.8)
Treatment Response, % (Increase in Hb ≥ 1 g/dL)	794 (72.1%)

**Table 3:** Comparison of treatment efficacy parameters in subgroups.

	Δ Hemoglobin (±SD), g/dL	*p*	Response Rate, %	*p*
Gender		0.87		0.018
Female	1.81 (±1.27)		74.5%	
Male	1.79 (±1.62)		67.7%	
Age		0.35		0.70
<65	1.83 (±1.41)		72.4%	
≥65	1.75 (±1.41)		71.4%	
Tumor Type		0.67		0.41
Gastrointestinal	1.76 (±1.32)		72.9%	
Breast	1.83 (±1.48)		69.9%	
Genitourinary	1.63 (±1.49)		68.4%	
Lung	2.09 (±1.67)		77.0%	
Gynecological	1.92 (±1.29)		74.2%	
Hematological	1.73 (±1.66)		61.2%	
Head and Neck	2.6 (±1.23)		88.5%	
Other	1.81 (±1.88)		74.1%	
Anemia Grade		<0.005		<0.005
Grade 1	1.52 (±1.24)		68.1%	
Grade 2	2.39 (±1.53)		79.4%	
Grade 3	3.06 (±1.66)		93.7%	
Active Treatment		0.04		0.24
Yes	1.74 (±1.30)		72.9%	
No	2.01 (±1.73)		69.0%	

**Table 4:** Baseline clinical and hematological characteristics of patients with gastrointestinal cancers.

	N = 510
Age (years, median (Q1–Q3))	57 (45–68)
Female, *n* (%)	252 (49.4%)
Cancer type, *n* (%)	
Upper GI	147 (28.8%)
HPB	80 (15.7%)
Lower GI	283 (55.5%)
Active chemotherapy, *n* (%)	423 (82.9%)
Baseline hemoglobin	
Mean (SD)	10.4 (±1.7) g/dL
Median (Q1–Q3)	10.7 (9.8–11.4)
Δ hemoglobin	
Mean (SD)	1.76 (±0.34) g/dL
Median (Q1–Q3)	1.60 (0.9–2.7)
Treatment response, % (increase in Hb ≥ 1 g/dL)	372 (72.9%)

**Table 5:** Comparison of treatment efficacy parameters among GI cancer patients.

	Δ Hemoglobin (±SD), g/dL	*p*	Response Rate, %	*p*
Gender		0.81		0.21
Female	1.77 (±1.22)		74.6%	
Male	1.74 (±1.52)		69.2%	
Age		0.87		0.54
<65	1.77 (±1.35)		72.1%	
≥65	1.75 (±1.26)		74.6%	
Tumor Location		0.91		0.40
Upper GI	1.80 (±1.36)		72.1%	
HPB	1.78 (±1.32)		67.5%	
Lower GI	1.74 (±1.30)		74.9%	
Active Treatment		0.03		0. 04
Yes	1.71 (±1.25)		72.9%	
No	2.04 (±1.57)		84.0%	

## Data Availability

Data are available upon request.
